# Utilization of Nutritional Supplementation Services and Their Predictors in Pregnant and Lactating Women Living in an Urban Resettlement Colony of Delhi, India: A Cross-Sectional Study

**DOI:** 10.7759/cureus.32302

**Published:** 2022-12-07

**Authors:** Sumi Paul, Saurav Basu, Baani Sodhi, Mongjam M Singh

**Affiliations:** 1 Indian Institute of Public Health-Delhi, Public Health Foundation of India, Delhi, IND; 2 Community Medicine, Maulana Azad Medical College, Delhi, IND

**Keywords:** lactation, nutrition education, nutrition assessment, nutrition support, pregnancy surveillance, nutrition status, nutrition policies, india

## Abstract

Introduction

Maternal undernutrition during pregnancy and lactating has adverse health consequences for the mother and her child. The Integrated Child Development Services (ICDS) scheme by the Government of India provides supplementary nutrition services to all pregnant and lactating women but its utilization is suboptimal due to inefficient distribution through the health system and beneficiary concerns regarding its usefulness.

This study was conducted with the objective of assessing the utilization of nutrition-related ICDS services by pregnant and lactating women in urban poor settlements of Delhi and the sociodemographic factors associated with non-utilization.

Materials and methods

This was a community-based cross-sectional survey in an urban resettlement colony and slum area located in the northeast district of Delhi. The data collection was conducted from January to May 2022. Eligible participants included pregnant women in their second or third trimester of pregnancy and lactating mothers in their first six months of the postpartum period who were residents of the study setting. Data were collected using face-to-face interviews using a pre-tested and self-designed questionnaire. The primary outcome was the proportion of women utilizing ICDS take-home rations (THR) in the previous month.

Results

A total of 365 participants were recruited in this study including 208 pregnant and 157 lactating women having a median (IQR) age of 25 (22-28) years. A total of 211 (57.8%) participants reported a history of utilization of ICDS supplementary nutrition services during their current pregnancy or postpartum with 154 (42.2%) having received THR in the previous month. Among the currently pregnant women, 84 (40.4%) had utilized ICDS THR while in the currently lactating women, 70 (44.6%) had utilized THR in the previous month. On adjusted analysis, multigravid women were less likely to have utilized ICDS compared to primigravida women.

Reasons for non-utilization of the ICDS supplementary nutrition services by the mothers were temporary disruption due to cessation of ICDS services by protesting Anganwadi workers (over demand for increased honorarium), difficult access to Anganwadi center, the poor perceived taste of the food provided as THR, and perception by the mothers that they did not require THR. Most women reported sharing the THR with their family members.

All pregnant and lactating women were found to be calorie and protein deficient in this study when applying the recommended intake values. No association was observed between the utilization of ICDS in the previous month and the presence of calorie deficiency in both pregnant (p=0.35) and lactating (p=0.24) women.

Conclusions

More than four in ten eligible pregnant and lactating women in an urban resettlement colony in Delhi did not utilize ICDS supplementary nutrition services with similar rates of utilization in both pregnant and lactating women. Women living in joint families and consequently larger households were less likely to utilize ICDS services. A majority of pregnant and lactating women were calorie and protein deficient even on applying non-pregnant cut-off requirements. The ICDS scheme needs to strengthen information, education, and communication (IEC) strategies and interventions to improve its acceptability and utilization by this vulnerable population.

## Introduction

Maternal undernutrition is a major public health challenge in India with a majority of Indian women entering pregnancy having anemia and nearly a third having low body mass index (BMI) [1,2]. Poor nutrition during pregnancy increases the risk of adverse pregnancy outcomes. Furthermore, nutritional inadequacy during lactation is highly detrimental to fetal and child growth during the crucial first 1000 days of life that involve the development of the brain, immune system, and overall growth [2-4]. To improve maternal nutrition status, healthy eating, nutritional counseling, and additional weight gain monitoring are recommended [5].

The Integrated Child Development Services (ICDS) scheme launched in 1975 by the Government of India (GOI) is one of the largest community-based interventions to improve the health and nutritional status of under-five children and pregnant and lactating women. The ICDS prescribes norms for the provision of daily supplementary nutrition equivalent to 600 kcal and 18-20 grams of protein free of cost to pregnant and lactating women through Anganwadi centers (AWCs), a type of rural government-primarily child care center existing in India. Nutrition and health education at AWCs is provided by female staff known as Anganwadi workers (AWWs). Currently, after the Covid-19 pandemic, take-home rations (THR) instead of hot cooked meals are being provided as part of the ICDS Supplementary Nutrition Programme (SNP) to all eligible beneficiaries including pregnant and lactating women [6].

Nevertheless, despite the efforts taken by GOI, the utilization of Anganwadi services among pregnant women in India remains suboptimal [7-12]. A large nationally representative cross-sectional survey (2015-16) reported that 46% of the pregnant and 51% of the lactating women did not receive any ICDS service [13]. A study in Northern India reported lactating women utilize the ICDS services at a higher rate as compared to pregnant women [10].

The Covid-19 pandemic had significantly disrupted national health programs in India including the ICDS due to lockdowns, fear of infection in the public, and diversion of frontline workers for other Covid-related duties which potentially compromised the sustainability of existing food systems, especially amongst vulnerable communities [14]. However, there is a paucity of information on the utilization of ICDS in Northern India during the period of the Covid-19 (THR) pandemic.

Therefore, this cross-sectional survey was conducted with the objective of assessing the utilization of nutrition-related ICDS services by pregnant and lactating women in urban poor settlements of Delhi and the sociodemographic factors associated with non-utilization.

## Materials and methods

Study design and setting

This was a community-based cross-sectional survey in an urban resettlement colony and slum area located in the northeast district of Delhi. The data collection was conducted from January to May 2022.

Study population

Pregnant women in their second or third trimester of pregnancy and lactating mothers in their first six months of the postpartum period who were residents of the study setting were included in this study.

Study tools and measurements

A self-designed questionnaire was developed in consultation with two public health experts who had considerable service experience in the study area. It included three sections prepared in English and translated into the local language Hindi was used to collect data from the study participants. The first section collected data on patient demographics, awareness about Anganwadi services, and gravida, parity, live birth, and abortion (GPLA). The second section collected data from the women on their utilization of ICDS services which included the information on utilization of supplementary nutrition which was being provided through THR supplements along with a brief assessment of their dietary and nutritional awareness. The questionnaire was pretested in 10 women to assess comprehension and for further refinement of the tool and administered by a female investigator. We did not ascertain the functioning of other ICDS benefits such as immunization, iron-folic acid supplementation, and growth monitoring because these functions were primarily implemented at the urban primary health facility in the area by public health nursing and medical practitioners.

Operational definitions

The 24-hour food recall data collected from the participants was then estimated to assess calorie and protein intake for the day which was further used to assess the calorie and protein deficiency in the participants. The minimum calorie requirement was considered as 2250 (1900 + 350) kcal/day for pregnant women, and 2500 (1900 + 60) kcal/day for lactating women. The minimum protein requirement was calculated as body weight in kg * 0.83 g/kg/day + (7 g/kg/day if in the second trimester) or (23 g/kg/day if in the third trimester) or (19 g/kg/day if lactating) [15].

Sample size and sampling strategy

The sample size required for the study was calculated using the prevalence of utilization of ICDS among pregnant women in urban areas (primary outcome) which was considered 38.8% from the National Family Health Survey (NFHS) (2015-16) [13], with 5% absolute precision and 10% non-response rate, the final sample size of 365 was calculated. Consecutive sampling was used to enroll at least 10 women in a single day from the community. The assistance of frontline female link workers known as Accredited Social Health Activists (ASHAs) was used to identify the pregnant and lactating women in the community.

Data management and statistical analysis

A validated database with inbuilt checks, designed using Census and Survey Processing System (CSPro Version 7.5) software (United States Census Bureau, Maryland, USA), was used for the single data entry. The data was entered and analyzed using the statistical software Stata (StataCorp. 2017. Stata Statistical Software: Release 15. College Station, TX: StataCorp LLC). Categorical variables were reported as frequency and proportions. A chi-square test was done to assess the factors associated with the utilization of ICDS. A binary logistic regression analysis was conducted to identify the factors independently associated with the utilization of ICDS. A p-value <0.05 was considered statistically significant.

Ethical considerations

The institutional ethics board at the Indian Institute of Public Health-Delhi approved the study protocol before the commencement of the study. Participants were provided with a detailed Participant Information Sheet. Those who consent to participate and sign the consent form were only included in the study. Confidentiality was maintained throughout the study by giving a unique ID instead of recording their names. The collected data was only used for research purposes.

## Results

A total of 365 participants were recruited in this study including 208 pregnant and 157 lactating women having a median age of 25 years. A majority of the women had completed intermediate education (61.9%) but only 0.8% of the women were employed. Nearly four in five women were living in joint families (81.6%). Almost all (98.9%) participants were aware of the Anganwadi services. The sociodemographic characteristics of the participants are reported in Table 1.

**Table 1:** Sociodemographic characteristics of the participants ^a^ Gravida and live children status missing for 102 and 100 participants, respectively ^b^ ASHA: Accredited Social Health Activist; ANM: Auxiliary Nurse Midwife

Characteristic	Frequency (%) (N=365)
Age (years)	
<25	142 (38.9)
≥25	223 (61.1)
Mother’s education status (years)	
<12	139 (38.1)
≥12	226 (61.9)
Husband’s education status (years)	
<14	172 (47.1)
≥14	193 (52.9)
Type of family	
Nuclear	67 (18.3)
Joint	298 (81.6)
Occupation of mother	
Housewife	362 (99.1)
Working	3 (0.8)
Occupation of husband	
Employed	363 (99.4)
Unemployed	2 (0.5)
Religion of the mother	
Hindu	357 (97.8)
Others	8 (2.1)
Per capita income	
<12000	147 (40.2)
≥ 12000	218 (59.7)
Total family members	
<6	159 (43.5)
≥6	206 (56.4)
Possession of Maternal Child Protection card	365 (100.0)
Awareness of Anganwadi services	
Yes	361 (98.9)
No	4 (1.1)
Gravida (N=263)^a^	
Primi	55 (20.9)
Multi	208 (79.1)
Number of children (N=265)^a^	
None	145 (54.7)
≥1	120 (45.2)
Visit by ASHA^b^	
ANM	42 (13.7)
ASHA	23 (38.3)
Both	263 (86.2)
No one	37 (61.6)

A total of 211 (57.8%) participants reported a history of utilization of ICDS supplementary nutrition services during their current pregnancy or postpartum with 154 (42.2%) having received THR in the previous month. Among the currently pregnant women, 84 (40.4%) had utilized ICDS THR while in the currently lactating women only 70 (44.6%) had utilized THR in the previous month.

Reasons for non-utilization of the ICDS supplementary nutrition services reported by the mothers were temporary disruption due to cessation of ICDS services by protesting AWWs, difficult access to AWC, the poor perceived taste of the food provided as THR, and perception by the mothers that they did not require THR. Most (98.17%) women reported sharing the THR with their family members.

On bivariate analysis, women living in joint families having a larger family size (>=6), and multigravida women reported lower utilization of ICDS services compared to those living in nuclear families and those having a smaller family size and these differences were statistically significant (Table 2).

**Table 2:** Distribution of factors associated with utilization of ICDS supplementary nutrition in pregnant and lactating women ICDS: Integrated Child Development Services; THR: take-home ration

Characteristic	No. of observations	THR received at least once n (%)	None n (%)	p-value
Age (years)				0.37
<25	142	64 (45.1)	78 (54.9)	
≥25	223	90 (40.3)	133 (59.6)	
Mother’s education status (years)				0.93
<12	139	59 (42.4)	80 (57.5)	
≥12	226	95 (42.1)	131 (57.9)	
Husband’s education status (years)				0.90
<14	172	72 (41.8)	100 (58.1)	
≥14	193	82 (42.4)	111 (57.5)	
Type of family				0.06
Nuclear	67	35 (52.2)	32 (47.7)	
Joint	298	119 (39.9)	179 (60.1)	
Religion of the mother				0.01
Hindu	357	147 (41.1)	210 (58.8)	
Others	8	7 (87.5)	1 (12.5)	
Socio-economic status				0.28
<12000	147	67 (45.5)	80 (54.4)	
≥12000	218	87 (39.9)	131 (60.1)	
Total family members				0.03
<6	159	77 (44.4)	82 (51.5)	
≥6	206	77 (37.3)	129 (62.6)	
Awareness of Anganwadi services				0.43
Yes	4	1 (25.0)	3 (75.0)	
No	361	153 (42.3)	208 (57.6)	
Type				0.42
Pregnant women	208	84 (40.3)	124 (59.6)	
Lactating women	157	70 (44.5)	87 (55.4)	
Gravida				0.004
Primi	55	34 (61.8)	21 (38.1)	
Multi	208	84 (40.3)	124 (59.6)	
Number of children				0.01
None	57	34 (59.6)	23 (40.3)	
≥1	208	84 (40.3)	124 (59.6)	

Variables showing a significant association with the utilization of ICDS THR in the bivariate analysis were included in the logistic regression analysis. On adjusted analysis, the variable multigravida in pregnant women had 0.38 times decreased odds of receiving THR when compared to the primigravida. The number of children variable was omitted from the multivariable regression analysis because of collinearity. Hosmer and Lemeshow test was performed to assess the goodness of fit of the model (p=0.12) and was obtained (p-value >0.05 indicates a good fit) (Table 3).

**Table 3:** Regression analysis to predict the factors associated with utilization of ICDS ^a^ Gravida and live children status missing for 102 and 100 participants, respectively ^c ^The variable number of children was omitted because of collinearity cOR: crude odds ratio; aOR: adjusted odds ratio; 95% CI: 95% confidence intervals; ICDS: Integrated Child Development Services

Characteristic	cOR, 95% CI	aOR^a^, 95% CI
Type of family		
Nuclear	Ref	Ref
Joint	0.61 (0.35, 1.03)	0.85 (0.45, 1.59)
Religion of the mother		
Hindu	Ref	Ref
Others	10 (1.21, 82.14)	3.5 (0.35, 35.62)
Total family members		
<6	Ref	Ref
≥6	0.63 (0.41, 0.96)	0.64 (0.38, 1.09)
Gravida		
Primi	Ref	Ref
Multi	0.41 (0.22, 0.77)	0.38 (0.21, 0.71)
Number of children^c^		NA
None	Ref	
≥1	0.45 (0.25, 0.83)	

Deficiency in calories was assessed using two cut-off values, one the standard cut-off value for sedentary women (1900 kcal) and the other inclusive of the additional calorie requirement for both pregnant (2250 kcal) and lactating (2500 kcal) women. Amongst lactating women with the standard cut-off value for sedentary women (1900 kcal), the mean (SD) calorie deficiency was -82.7 (62.5) and out of 157 participants, 102 (64.9%) were reported to be calorie deficient. With the recommended calorie cut-off (2500 kcal), the SD calorie deficiency was -514.5 (78.7) and all 157 participants were found to be calorie deficient. Among the pregnant women using the standard cut-off (1900 kcal), the SD calorie deficiency was estimated as -73.0 (60.6) with 91 (43.7%) out of 208 participants observed to be calorie deficient. On applying the recommended calorie cut-off (2250 kcal), the SD calorie deficiency was -519.3 (81.9) with all the pregnant participants observed to have calorie deficiency.

Similarly, in lactating participants, the SD protein intake using the 24-hour recall method was 71.0 (9.1) gm and all 157 lactating participants were observed to have protein deficiency. Among the pregnant women, the SD protein intake using the 24-hour recall method was 70.6 (8.5) gm. On estimating the average weight of the women as per the reference Indian women standard (55 kg), all 208 pregnant women were found to be protein deficient. No association was observed between the utilization of ICDS in the previous month and the presence of calorie deficiency in both pregnant (p=0.35) and lactating (p=0.24) women.

All of the women recognized the importance of diet and nutrition during pregnancy and childbirth but awareness of specific food groups was low, while one in four women was unaware of the benefits of diversifying their dietary consumption (Table 4).

**Table 4:** Awareness related to nutrition and diet

Items related to awareness of diet and nutrition	n (%) (N=365)
Diet is important during pregnancy	365 (100.00)
Right type and amount of nutrition during pregnancy and lactation is crucial for the growth of the unborn and breastfed baby	365 (100.00)
Any five essential groups of food important for the growth of the baby (n=364)	239 (65.66)
Benefits of consuming variety of foods during pregnancy and lactation (n=364)	273 (75.00)
Well balanced diet supports two lives (n=364)	364 (100.00)
Heard about carbohydrates	46 (12.60)
Heard about vitamins	76 (20.82)
Heard about minerals	72 (19.73)

## Discussion

This study examines the utilization of supplementary nutrition provided under ICDS services to pregnant and lactating in an urban resettlement and slum colony area in Delhi, India. The study observed that more than half of the eligible beneficiaries were not utilizing supplementary nutrition-related ICDS services. Lower rates of utilization of ICDS services by urban pregnant (38.8%) and urban lactating (35.6%) women were reported in NFHS-4 (2015-16) while the NFHS (2019-20) reported overall 66% of pregnant and lactating women as utilizing ICDS supplementary nutritional services [13,16]. Our study findings corroborate findings from previous studies suggestive of low utilization of ICDS supplementary nutrition services, with a higher proportion of lactating compared to pregnant women utilizing these services [7-12].

A study from Western India reported multiple factors related to household convenience and accessibility of ICDS services influenced their utilization by mothers [17]. Another study from Southern India in Ernakulam district reported that lack of time, improper communication with AWWs, poor behavior of AWWs, the distance of AWC from home, and lack of knowledge were the main barriers restricting the usage of Anganwadi nutrition services [18]. A study from Eastern India in the Ganjam District reported the association of factors like socioeconomic status (SES), literacy, and occupation with the extent of ICDS utilization [12].

In this study, all pregnant and lactating women were calorie deficient when considering the recommended intake with no association observed with ICDS utilization. A study conducted in the Siddipet district of Telangana in the Eastern coast of India reported that 90% of pregnant and 50% of lactating women were not consuming adequate amounts of calories and proteins [7]. The findings of another study conducted in the Medak district also observed significantly reduced protein and energy intake amongst pregnant and lactating women attending ICDS services [19].

The present study did not observe any statistically significant association between SES and ICDS utilization in contrast to previous studies that observed underutilization of ICDS by women belonging to high SES households probably due to the overall low-income setting of our study [8,17]. This study observed no association of education with ICDS utilization although there is mixed evidence in this regard [8,17]. Similar to our study, Sabat and Karmee (2021) in the Eastern state of Odisha reported a universal preference for sharing of ICDS THR by female beneficiaries with their family members, suggestive of a common cultural practice prevalent across diverse geographies in India [12].

Our study has a few limitations. Firstly, the outcome variable collecting the data on the utilization of supplementary nutrition from ICDS was based on recall and could not be objectively verified which can contribute to information bias. The data collection coincided with the onset and progress of the Omicron wave of the Covid-19 pandemic that could have reduced utilization of ICDS by the beneficiaries although, in this study, none of the women reported fear of infection as a reason for non-utilization of ICDS. However, some periods of the data collection period coincided with a protest by AWWs demanding an increase in honorarium resulting in the disruption and inability to access ICDS services by some of the beneficiaries [20]. Consequently, this study is likely to have underestimated the utilization of ICDS in pregnant and lactating women in the area. Secondly, the use of 24-hour dietary food recall was a limitation, since it cannot account for day-to-day variation in diet. The long-term dietary habits of the study participants cannot be assessed using this method either [21]. Household food security was not assessed in this study but it is an important determinant of calorie and protein intake whose deficiency is a determinant of maternal undernutrition. Lack of prospective data collection precluded a temporal assessment of the effectiveness of ICDS on the reduction of nutritional deficiency in the participants, and neither could we assess the persistence of the use of ICDS services during the course of pregnancy and lactation. Finally, this survey was limited to the assessment of ICDS supplementary nutrition services and did not assess other functions of the program such as immunization and growth monitoring.

## Conclusions

More than four in ten eligible pregnant and lactating women in an urban resettlement colony in Delhi did not utilize ICDS supplementary nutrition services with similar rates of utilization in both pregnant and lactating women. Most pregnant and lactating women were calorie and protein deficient indicative of socioeconomic vulnerability but it was not associated with ICDS utilization suggestive of the inadequacy of this pivotal long-standing public health intervention in protecting women from consuming impoverished diets. The ICDS scheme needs to strengthen information, education, and communication (IEC) strategies and interventions to improve its acceptability and utilization by the target population to realize sustainable development goals.
